# Exploring women’s exposure to marketing of commercial formula products: a qualitative marketing study from two sites in South Africa

**DOI:** 10.1080/16549716.2022.2074663

**Published:** 2022-08-10

**Authors:** Catherine Pereira-Kotze, Christiane Horwood, Lyn Haskins, Gillian Kingston, Silondile Luthuli, Tanya Doherty

**Affiliations:** a School of Public Health, University of the Western Cape, Cape Town, South Africa; b Centre for Rural Health, University of KwaZulu-Natal, Durban, South Africa; c M&C Saatchi World Services, King’s College, London; d Health Systems Research Unit, South African Medical Research Council, Cape Town, South Africa

**Keywords:** Promotion, advertising, breast-milk substitutes, infant and young child feeding, South Africa, legislation

## Abstract

**Background:**

Regulating the marketing of commercial formula products is a long-term commitment required to protect breastfeeding. Marketing strategies of formula manufacturers, retailers and distributors evolve at a rapid rate.

**Objective:**

The aim of this research was to describe exposure of pregnant women and mothers of young children in South Africa to marketing of commercial formula products, compared to international recommendations and national legislation.

**Methods:**

Using mobile phone marketing diaries twenty participants in Cape Town and Johannesburg documented the formula marketing they were exposed to for one week. Ten mothers were interviewed to explore their perceptions towards marketing exposure in more depth.

**Results:**

Women reported limited infant formula advertising, but an abundance of strategies used to market growing-up formula and powdered drinks for children over 36 months. Strategies included product packaging, in-store displays, online distribution channels and educational material about product ranges. Online strategies were reported, namely social media marketing (sponsored adverts and support groups), websites and mobile phone applications providing infant and young child feeding information and price discounts, print and TV advertisements, and competitions. Products for children over 36 months are cross-promoted with products prohibited to be advertised by national legislation.

**Conclusions:**

South African women are being exposed to covert marketing of infant, follow-up, and growing-up formula. Explicit marketing of products for children over 36 months of age allows formula companies to provide messages about branding and use of commercial formula products to mothers. National legislation should be updated and effectively implemented to address changing marketing strategies.

## Background

Regulating the marketing of commercial formula products is a long-term commitment that can contribute to the protection of breastfeeding, which is the foundation of optimal infant and young child feeding (IYCF). Breastfed children have better health and development outcomes compared to children who are not breastfed, with lifelong benefits. Exclusive and sustained breastfeeding, in accordance with World Health Organization (WHO) guidelines, is associated with reduced morbidity from common childhood diseases, improved growth, higher intelligence, and long-term higher achievement [1]. Longer periods of breastfeeding also benefit mothers, by increasing birth spacing and reducing breast cancer risk [1]. In addition breastfeeding is environmentally sustainable, avoiding the catastrophic environmental impact of large scale formula manufacturing [2]. Despite these well-recognised benefits, breastfeeding rates are still very low, globally and in South Africa [3,4].

The International Code of Marketing of Breast-milk substitutes (known as the Code), adopted by the World Health Assembly in 1981, seeks to regulate the marketing of commercial formula products and remove marketing pressures from decision making about infant feeding, to contribute to the protection of breastfeeding [5]. The Code defines marketing as a broad concept that includes *‘product promotion, distribution, selling, advertising, product public relations and information services’* [5]. Accepting that distribution and selling of commercial formula products is necessary, restrictions are primarily aimed at the promotion and advertising of these products as well as the provision of information on IYCF and nutrition. Marketing is considered to be a structural determinant influencing the environment for breastfeeding [6]. Marketing of commercial formula products has a negative impact on breastfeeding practices [7]. Marketing strategies of manufacturers, retailers and distributors of commercial formula have evolved over the four decades since the Code was ratified towards marketing approaches designed to evade the restrictions, including through social media [8,9].

South Africa gazetted the regulations relating to foodstuffs for infants and young children (R991) in 2012 in terms of the Foodstuffs, Cosmetics and Disinfectants Act (No. 54 of 1972) as the mechanism to legislate the Code in South Africa [10]. R991 is considered to be substantially aligned with the Code [11] and regulates the marketing of all products for infants and children under 36 months of age including all commercial formula products, complementary foods, bottles and teats [10]. There are also provisions restricting information on IYCF and nutrition in South Africa, but these do not clearly describe the type of content that is prohibited on information, educational and communications materials [11]. The regulations are not consistently implemented and enforced, and violations are taking place frequently [12].

The aim of this study was to describe the exposure of pregnant women and mothers of young children in South Africa to the marketing of commercial formula products as compared to international codes of practice and current national legislation.

## Methods

### Study design

This research formed part of a larger multi-country mixed-method study that used consumer and market research methods to explore how women and mothers make decisions regarding IYCF [13]. This qualitative component documented the exposure of pregnant women and mothers of infants and young children aged 0–18 months in South Africa to the different marketing practices used by manufacturers, distributors, and retailers of commercial formula products. Data were collected using mobile phone marketing diaries and in-depth interviews (IDIs). An ethnographic approach was used during IDIs to document women’s lived experiences exposure to formula marketing [14].

### Study sites and sampling

For the phone marketing diaries, 20 pregnant women and mothers of children aged < 18 months were recruited from the largest cities in South Africa, Cape Town, and Johannesburg, using convenience sampling. Varying sample sizes are used in diary studies, but for the purposes of this research, it was felt that 20 participants would enable data saturation based on our knowledge of the content area (infant formula marketing) and a review of similar studies [15,16]. A combination of on-street recruitment and snowball sampling (where women recommended a friend to take part) were used. Potential participants were asked to read several eligibility screening questions before being included in the sample. These included questions to evaluate literacy and ensure that respondents would be able to read written messages on product packaging or advertisements and complete a phone marketing diary themselves. Ten of the 20 women who participated in the phone marketing diaries were purposively selected to participate in an individual IDI, based on having a variety of different feeding practices and willingness to participate. All IDI participants were mothers; pregnant women were not included in IDIs since the aim was to explore perceptions of marketing among women with a variety of current feeding practices. Five participants were selected from each city including mothers using different feeding practices (breastfeeding, mixed feeding or formula feeding). An initial review of the 20 marketing diaries and 10 IDI transcripts determined sufficient commonality and unique experiences. It was deemed not necessary to recruit further women.

### Participants and data collection

Data collection was conducted by an agency (KLA), who specialises in market research. All interviewers were female. For a period of one week, mothers were asked to record their exposure to marketing of commercial formula products (including infant formula, follow-up formula, liquid or powdered milks or powdered drinks marketed or otherwise represented as suitable for infants and young children). They were asked to take a photo or screenshot, every time they saw an advert for or picture of any product or brand that they considered to be a commercial formula product, or any time they received any formula promotion to their phone or email or on any of their social media applications (such as Facebook or Instagram). Images were shared via WhatsApp mobile phone instant messaging with a member of the research team and saved in a WhatsApp chat. Individual IDIs were approximately 90 minutes in length and were conducted in mothers’ homes so that mothers felt comfortable in their natural setting [14]. A semi-structured interview guide was used which included open-ended questions about the media exposure captured in the phone marketing diaries and its impact on participants attitudes to formula milk, as well as questions around infant feeding behaviours and attitudes. The researcher asked the mother to share what she had captured in the marketing diary and used the diary to elicit more in-depth discussion about the marketing of formula. Following each interview, the researcher took notes reflecting on the discussion and their own personal feelings and possible biases towards the issues discussed. Based on extensive experience of using the consumer marketing methodology, it was anticipated that collecting marketing diary information from 20 participants would provide sufficient breadth of exposure from different sources to provide a rich picture of mothers’ marketing exposure.

### Data analysis

Participants shared images of all marketing of commercial formula products they observed via WhatsApp. Characteristics of images were coded (in categories according to the manufacturer, brand, type of product marketing channel used, and the messaging or claims used) and entered into a Microsoft Excel spreadsheet. Data was categorised into products intended for children above and below the age of 36 months (i.e. those covered by the R991 regulations, and those products excluded from the regulation). WhatsApp transcripts from the 20 phone marketing diaries and IDI transcripts from the 10 ethnographic interviews were analysed in a similar way. Transcripts were read and coded to identify themes across all data. Themes were identified using a content analysis approach [17,18]. Examples that displayed similar marketing strategies or platforms were grouped together. Certain images and quotations were selected to be shared in the results. Analysis was conducted by two researchers (GK and CPK). Two researchers (GK and CPK), independently read and re-read the transcripts to familiarise themselves with the data. One researcher (GK) created the initial coding framework based on categories derived from the research questions. A second researcher (CPK) then developed the coding framework further and created themes linking the categories of data.

### Ethical considerations

Ethics approvals were granted by the WHO ethics review committee (approval number ERC 003235) and the South African Human Sciences Research Council (HSRC) ethics committee in August 2019 (approval number 3/24/07/19). Informed consent was obtained from all participants. Participants were informed that confidentiality would be maintained and they could stop participating at any time without explanation. Participants were also informed, and consent was obtained for audio recording of interviews.

## Results

These results describe the experience of pregnant women or mothers of young children in South Africa (Table 1) and their exposure to content on the marketing of commercial formula products and/or promotion of formula feeding. Twenty mobile phone marketing diaries (MD) were completed by seven pregnant women and 13 women with babies aged below 18 months, and 10 IDIs were conducted with mothers of children aged < 18 months during February and March 2020.

**Table 1.** Profile of participants.

	Pregnant women (N = 7)	Mothers of infants 0–5 months (N = 5)	Mothers of children 6–18 months (N = 8)	Total (N = 20)
Study site				
Cape Town (N = 10)	3	3	4	10
Johannesburg (N = 10)	4	2	4	10
IYCF practice				
Exclusive Breastfeeding	N/A	3	1	4
Breastfed then changed to Formula	N/A	1	5	6
Formula fed from birth	N/A	1	2	3
Race/ethnicity				
Black African	4	4	4	12
Mixed race	1	0	2	3
Indian	1	1	0	2
White/Caucasian	1	0	2	3

Content of the phone marketing diaries included mainly photographs and screenshots as well as some videos and WhatsApp transcripts. Several themes were identified from analysis of the phone marketing diaries and the IDIs. Participants were asked to report on exposure to commercial formula products and the results show clear differences between the marketing of products for children under 36 months and the marketing of products for children over 36 months.

### Socio-demographics of participants

All participants were female and between the ages of 27 and 36 years.

### Women’s exposure to the marketing of commercial formula products in South Africa

Most participants reported that infant formula (formula indicated for infants 0–6 months) is not being advertised or promoted in shops, at baby exhibitions or on social media networks. This lack of exposure to advertising was perceived by some women as lack of information on formula feeding. Mothers reported that they do not know where to access information on formula feeding. A Cape Town mother described that:

… there was very, very limited advertising, marketing, or info on baby formula, which I never picked up before. It’s only now when I’m looking for it. (Mother of a 2-month-old, Cape Town, IDI-C1)

Some women contrasted this with breastfeeding which was considered more acceptable and where more support was available. A Johannesburg mother described:

… if you can’t breastfeed, you know, because everyone is pushing breastfeeding and then if you can’t, you don’t get any support for it. Well, you feel very alone because there’s no support for you. There’s support for you if you breastfeed, because then everyone can tell you, you must latch and this and do this. But if you don’t breastfeed, there’s no-one that can help you, so everyone is like, you have to – breast is best. (Mother of a 17-month-old child, Johannesburg, IDI-J1)

Since participants were struggling to find infant formula advertising, they started to actively search for examples of advertisements during the week that they were recording their phone marketing diaries. Even then, they struggled to find adverts for infant formula. A Johannesburg mother explained:

No, I’m not seeing adverts [for infant formula] anywhere and trust me I went and was like how can I not? I’m fairly intelligent, how can I not see an ad anywhere? I looked everywhere on all social medias, there’s none, you put in the word baby formula hoping that an ad would pop up and you get nothing, in fact you get lot of Purity [baby food] and lot of Elizabeth Arden [Elizabeth Anne toiletries and non-food baby products] and nothing for what you are looking for. (Mother of a 4-month-old infant, Johannesburg, IDI-J2)

Some women expressed surprise that advertising of or providing information about or promotion of ‘baby formula’ for children under 36 months is illegal in South Africa. One participant described the experience of not finding information about formula feeding ‘eye-opening’ and described that she *‘ … can’t believe how difficult it is to get information on formula milk*’ *(Mother of a 17-month-old child, Johannesburg, IDI-J1)*. Some mothers felt that there should be more information available on formula feeding. When specifically asked whether formula companies should be allowed to advertise, some participants indicated that they thought advertising of formula should be allowed, because they perceived marketing activities as a source of information rather than as companies promoting their products:

So, if there were actually ads online, highlighting the best part of the milk, perhaps it would be easier for me to make my decision, instead of having to do extensive research online and falling back on the paediatrician for advice. (Mother of an 8-month-old infant, Cape Town, IDI-C3)

### Marketing strategies used by companies to advertise commercial formula products in South Africa

This section describes the marketing of commercial formula products for children under 36 months, which is regulated by South Africa’s R991 legislation. This group of products includes infant, follow-up and growing-up formula. Although few formal adverts for infant formula were reported, there were strategies documented by participants, some of which could be categorised as traditional marketing and others as novel tactics (e.g. digital marketing). These include the use of product packaging by manufacturers; the use of cross-promotion with marketing products for older children; various strategies by retailers; and the extensive use of social media to market commercial formula products and formula feeding.

### Product packaging used to market commercial formula products

Many participants reported that products with certain characteristics related to labelling/packaging (e.g. use of the word ‘Gold’, ‘Pro’ or ‘Opti’ in the brand name or the colour Gold on the tin) attracted them, because it was thought that these products were of better quality. One participant described that when she compared two brands of formula, differences in packaging led her to assume that one brand was of a lower quality than the other, even though both brands were produced by the same manufacturer. The packaging of the slightly more expensive brand was described by the mother to be more *‘eye-catching and attractive’* and this, together with her perception that the higher price indicated a better-quality product, positively influenced her desire to purchase the product (*Mother of a 12-month-old child in Cape Town, MD-C4*).

**Figure 1.**
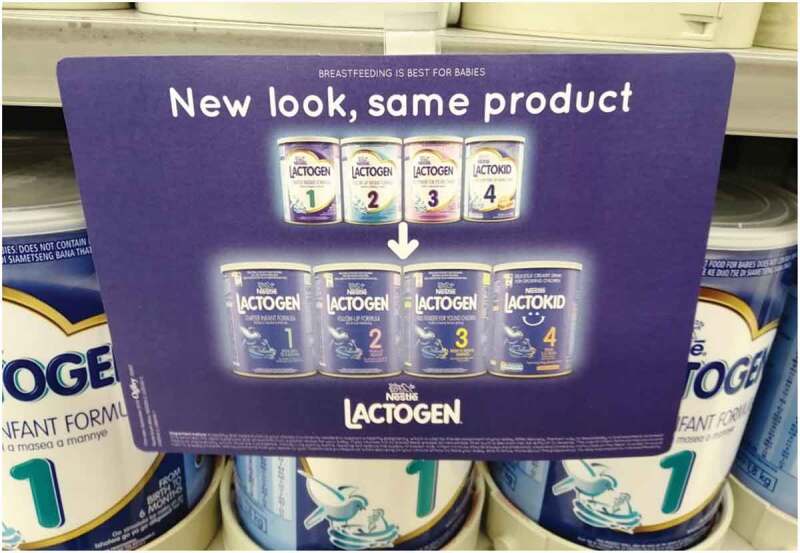
Example of cross-promotion between products intended for infants and young children of various age ranges shared by two Cape Town-based participants *(Pregnant woman, MD-C8; Mother of an 18-month-old, MD-C2).*

Product packaging was also used to cross-promote infant formula with products marketed for older children. Two participants shared the image in Figure 1, a shelf wobbler (or shelf talker) that describes new packaging for a range of commercial formula products. Cross-promotion can clearly be seen in both the existing and the new packaging, where the brand names, colour schemes, font type, layout of the packaging and imagery used on products intended for children under 36 months (infant, follow-up and growing-up formula – those with the numbers 1,2 and 3 on the tin) is very similar to that used for products indicated for children 36 months and older (those with the number 4 on the tin).

Some results show confusion and misunderstanding regarding whether infant formula can be given to an older child and if a product marketed for an older child can be given to a younger child. For example, the mother of a 12-week-old infant in Johannesburg *(IDI-J5)* displayed confusion regarding what the number ‘4’ on the tin referred to:

I think it said four months if I’m not mistaken. I remember seeing a 4 on the actual can but I don’t really know specifically what age it’s for.

### Retailer strategies to market commercial formula products

**Figure 2.**
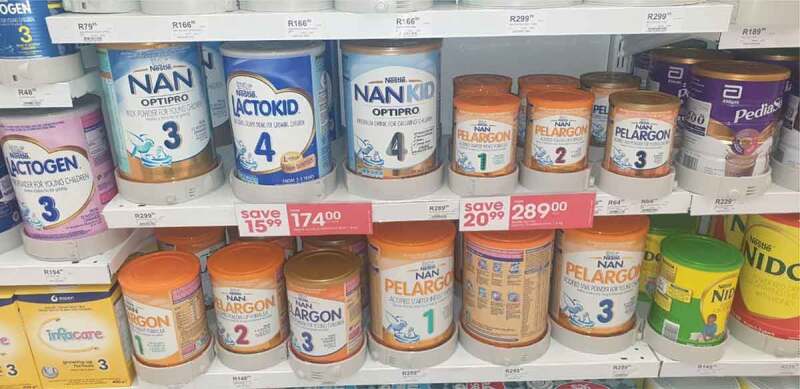
Price reductions and shelf wobblers drawing attention to infant, follow-up, and growing-up formulae at a pharmacy in Cape Town (*Mother of an 18-month-old child, MD-C2).*

The examples provided by participants show that retailers use a variety of strategies, both in-store and online, to advertise commercial formula products. In-store displays are used to market infant, follow-up, and growing-up formula. Participants shared examples of the use of shelf wobblers displaying information on product price to bring attention to commercial formula products, including those indicated for children under 36 months (Figure 2).

Another strategy used by retailers to market commercial formula products was the sharing of product information and educational material. Examples were provided by participants where women were shown information on product ranges (including products indicated for children under 36 months). Information was shared via pamphlets developed by various manufacturers and provided to retailers who then distributed this to mothers.

**Figure 3.**
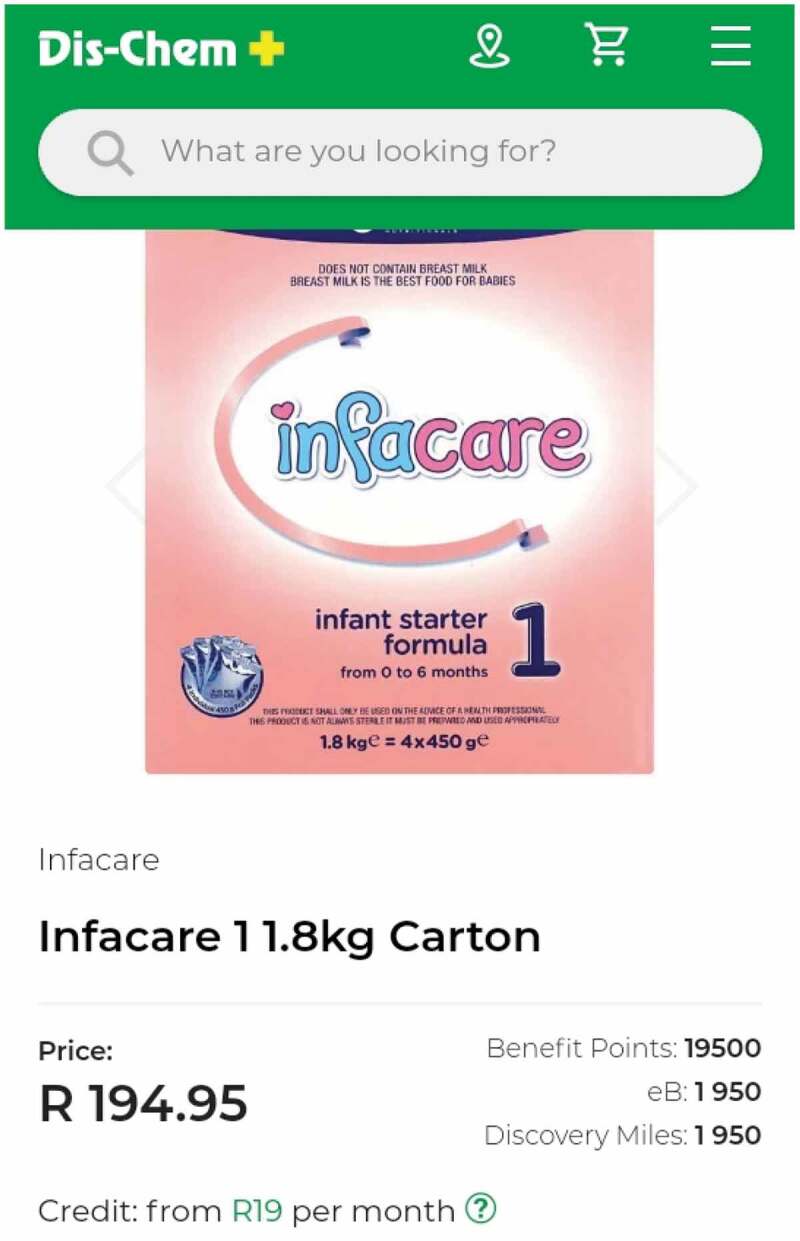
Examples of how loyalty points can be used to purchase infant formula online *(Mother of a 4-month-old infant, Johannesburg, MD-J2).*

Traditional marketing from retailers takes place in stores, however, retailers also appear to be using online distribution channels to market commercial formula products. The final example of retailer marketing practices is illustrated by two participants who shared examples in their phone marketing diaries of online retailers allowing infant formula and growing-up formula to be purchased using loyalty points that had been accumulated with the retailer. The examples in Figure 3 are from a pharmacy that retails online as well, which displays an infant formula that can be purchased using retailers’ loyalty points (Dis-Chem Benefit points), a bank’s loyalty points (FNB’s eBucks), a credit card’s loyalty points (Discovery Miles) or the option to purchase the infant formula on credit to be paid off over monthly instalments (Figure 3). However, while commercial formula products can be purchased using loyalty points, one participant described that the same loyalty points cannot be accumulated when infant formula is purchased:

I got a Dischem card, because there’s points back, although they told me you don’t get points for the baby formulas because they can’t have formulas on discounts, the only thing you don’t get points for are baby formulas and milk and medicine, those things are regulated by government, they don’t have a mark-up and whatever price is set, that’s it. (Mother of a 4-month-old infant in Johannesburg, IDI-J2).

The accrual of loyalty points at certain retailers appears to influence where consumers choose to shop. One mother was asked by the interviewer why she purchases infant formula from a particular store, and she responded:

The price range and … because you get that loyalty rewards. (Mother of a 12-month-old in Cape Town, IDI-C4)

### Women in South Africa are exposed to marketing of commercial formula products through various digital platforms including social media

Digital marketing techniques have evolved, and participants shared various examples of online marketing of commercial formula products. This content is produced by manufacturers of commercial formula products but also by retailers, social media users (consumers), online support groups and other sources. Some of this content is produced and shared in South Africa, but online users have access to content developed and shared in other countries. The types of content available online can be categorised according to information obtained from: search engines, social media (pages, groups, and sponsored posts), instant messaging applications, websites, email marketing, mobile phone applications and online distribution of products. Facebook, Instagram, and Twitter were described by participants as social media sites where the marketing of commercial formula products could be observed, with many participants expressing that the amount of content available online was overwhelming and sometimes contradictory.

#### Social media platforms used in marketing commercial formula

Sponsored ‘pop-up’ posts which are unsolicited content that appear on a user’s social media newsfeed, were described as common across various social media platforms. These demonstrate that the algorithms used by social media companies covertly promote commercial formula products. A Johannesburg mother shared the following:

Yes, the interesting part was like, so initially I wasn’t sure if she was getting enough milk and stuff so I tried to Google to see is there any recommended formulas or whatever. And then after I did that suddenly on my Facebook, Instagram, everywhere I would get pop ups on formula, which I never got before, but because I googled it, now suddenly it popped up everywhere, have you tried this, have you seen this … I was surprised at how quickly … if you just google it and then whatever search engine or app or whatever you use, it starts popping up. (Mother of a 3-month-old infant, Johannesburg, IDI-J5)

**Figure 4.**
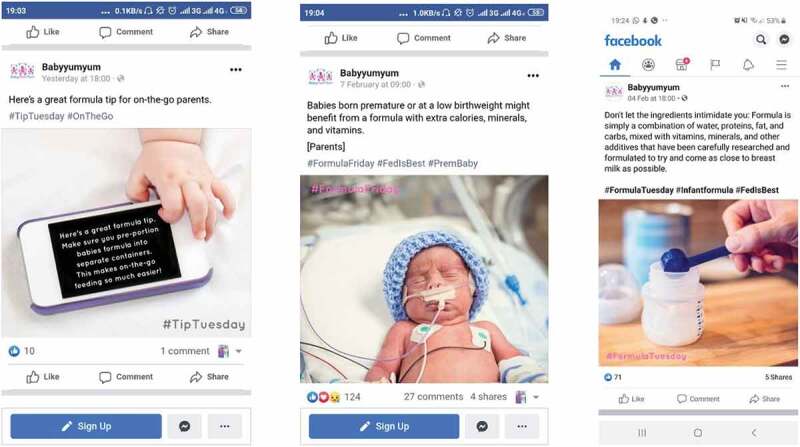
Examples of a Facebook page sharing information and content on formula feeding *(Mother of an 8-month-old infant, Cape Town, MD-C3; Mother of a 15-month-old child, Johannesburg, MD-J1).*

Facebook is a social media platform that has various ways of engaging with users. Women observed seeing marketing of commercial formula products on Facebook Pages and Groups (i.e. online support) that were either South African or international *(e.g. ‘Yummy Mummy’, ‘Mommy group Cape Town’ ‘Exclusive Breastfeeding and Pumping Support Group’; ‘First Time Mommies’; ‘Co-Sleeping and Attachment Parenting Support’; ‘Super Mommy’; ‘The Bump’; ‘Baby Center’; ‘Dis-Chem’; ‘Pampers’)*. Participants provided examples of Facebook pages and groups sharing content related to infant formula and products for children under 36 months ranging from ‘general tips’ to fairly emotive content. The images in Figure 4 were shared by two mothers based in Cape Town and Johannesburg and show different content from the same Facebook group including the use of the hashtag #FedisBest. This hashtag appears to be based on the commonly used phrase ‘*breast is best*’ but #FedisBest suggests that any type of feeding is best. While formula feeding is promoted and described, the same Facebook page was also found to share some content on breastfeeding.

#### Websites used in marketing of commercial formula

Participants shared numerous websites that provide content on IYCF. Several examples of South African sites were provided.^1^^1^https://www.huggies.co.za/articles/toddler/toddler-food/healthy-eating;http://www.inspiringwomen.co.za/everything-need-know-baby-formula/; https://babyyumyum.co.za/must-know-how-to-choose-a-baby-formula/;http://mamabythebay.com/2012/05/25/on-formula-failure-and-freedom/; https://babyyumyum.co.za/infant-formula-price-comparison/; https://www.babyandme.nestle.co.za/ An example was shared of a South African interactive website^2^^2^https://moomie.co.za/formula-and-bottle-feeding/243126-which-formula-to-start-newborn-baby-on#272060 where users post questions and receive answers/responses from other users. A few examples relating to formula feeding were provided, including a specific online conversation entitled ‘Which Formula to Start Newborn Baby On?’^2^ which illustrates the recommendation of a specific formula brand by various users.

**Figure 5.**
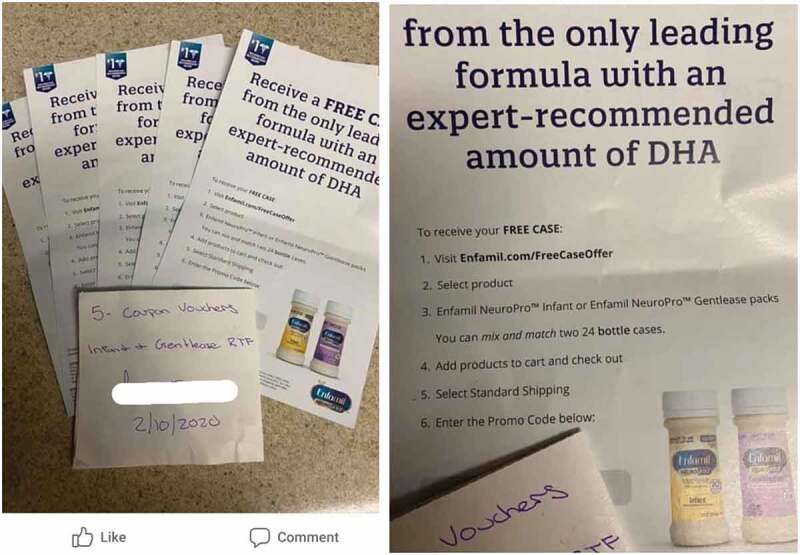
Example of flyers shared on an international Facebook group that enable mothers in the USA to access free samples of infant formula *(Pregnant woman, Cape Town, MD-C7).*

Mothers are also able to access international websites that contain information on formula. One participant shared an example of an American website^3^^3^https://www.babycenter.com/baby/formula-feeding/is-it-true-that-you-shouldnt-use-hot-tap-water-to-make-baby_10310200#:~:text=That%26s%20right.,pipes%20than%20cold%20water%20will that provides instructions on how to prepare ‘baby formula’. An example shared in a phone marketing diary displays clear promotion of infant formula (intended for use from 0–6 months) on a private Facebook group^4^^4^https://www.facebook.com/groups/419396622229188 in the USA with over 15 000 members (called ‘formula buy/sell/trade/bottles/diapers’) (Figure 5). Mothers can request to join this private group by answering a few simple questions. The participant was exposed to posts sharing vouchers for infant formula on this group in direct contravention of the Code.

#### Email newsletters used in marketing of commercial formula

Another route of exposure to content about commercial formula products is through email newsletters or mailing lists. One participant (a mother of a 2-month-old infant in Cape Town) shared a screenshot of an email about ‘feeding your baby’ that contained information on breastfeeding and formula feeding that she had received.

### Women’s exposure to the marketing of powdered drinks for children over 36 months

While our data shows that women had not been exposed to much overt or direct advertising of infant formula, many participants described seeing an abundance of adverts for ‘powdered drinks for growing children’ (i.e. products indicated for children older than 36 months). One mother described:

I noticed that from where I was, or what I was catching, I noticed more stuff for the older kids than for babies or little kids. (Mother of a 16-month-old in Johannesburg, IDI-J3)

**Figure 6.**
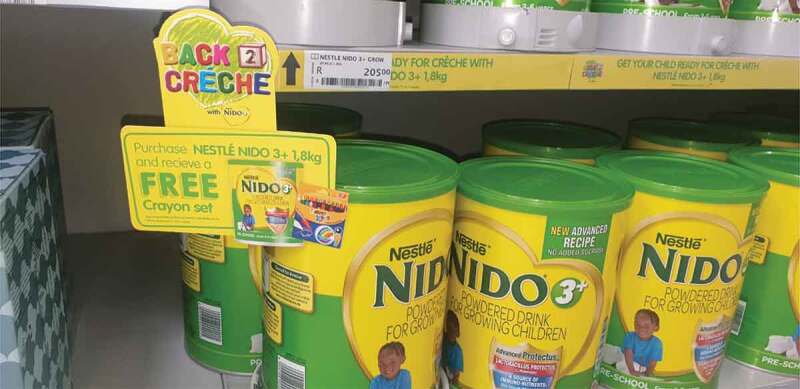
The mother of an 18-month-old child in Cape Town *(MD-C2)* shared an example of an in-store display of a product for children over 36 months.

Examples of promotion across different platforms were shared in the phone marketing diaries. As with infant, follow-up, and growing-up formula (products marketed for children under 36 months), some traditional marketing platforms were used, such as the use of product packaging, in-store promotion, newspaper supplements advertising price discounts and magazines for new parents with adverts containing claims about products for children over 36 months. In-store displays sometimes included the provision of a gift with the purchase of the product (Figure 6).

Several participants shared images of television adverts for ‘nutritional supplements’ or ‘powdered drinks for growing children’ indicated for use over the age of 3 years, that included examples of promotion such as endorsement from a health professional (e.g. ‘#1 Paediatrician Prescribed’) and negative nutrient claims (e.g. ‘no added sucrose’).

**Figure 7.**
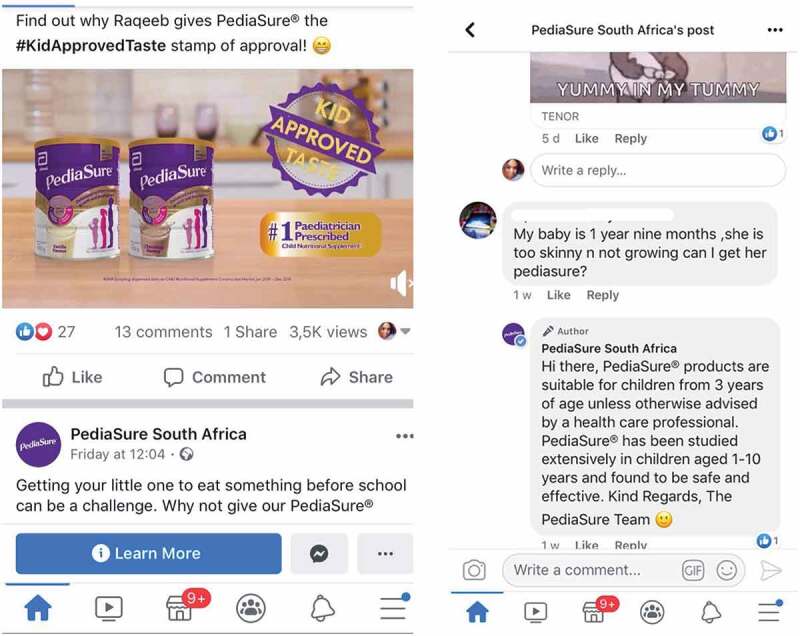
Facebook post of a product indicated for children over 36 months being advised for use under 36 months *(Pregnant woman, Cape Town, MD-C6).*

There were many examples on social media (e.g. Facebook) of content being shared related to products marketed to children over 36 months. The example in Figure 7, however, shows how a manufacturer has extended the age range of a product indicated for use over 36 months, to imply that it is suitable for use in children under the age of 36 months.

**Figure 8.**
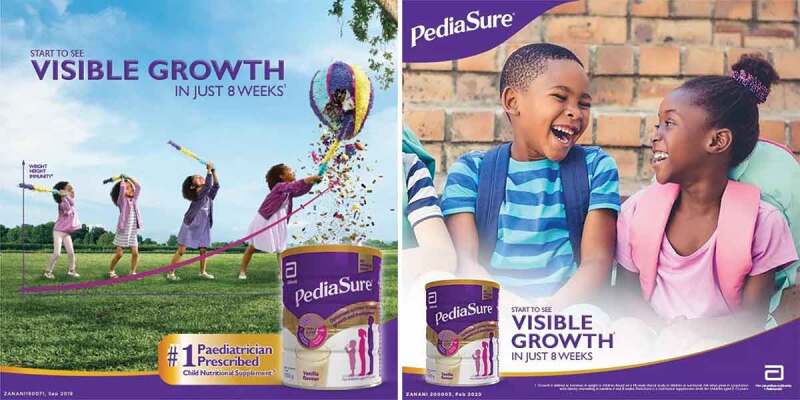
Facebook advertisements for a product for children over 36 months *(Pregnant woman, Cape Town, MD-C8).*

Many participants reported seeing advertisements for a ‘child nutritional supplement’ (indicated for use over 36 months) on social media as shown in Figure 8. These adverts include claims such as ‘visible growth in just eight weeks’ and ‘#1 Paediatrician Prescribed’.

## Discussion

This study shows that manufacturers, retailers, distributors of commercial formula products and others are using a plethora of marketing strategies to reach pregnant women and mothers. Traditional marketing such as in-store displays, product information leaflets and product packaging are still being used, together with more covert marketing techniques such as digital marketing, cross-promotion with products for older children and indirect contact with women through social media and online groups. Mothers report being confused by the variety of commercial formula products available to choose from, and when or how they should be used. In South Africa, despite having legislation to regulate marketing, manufacturers continue to promote commercial formula products. Although the R991 regulations have reduced formal advertising of formula products for children below 36 months, the regulations fail to control other marketing approaches to promote products for younger children.

Mothers in this study perceived there to be a lack of information available on formula feeding. The R991 regulations prohibit commercial entities from providing information about IYCF; only health care providers and professional associations can develop generic educational material on infant and young child nutrition, and health care personnel can demonstrate infant formula preparation to mothers who need to use it on an individual basis [10,19]. However, mothers are searching for information on infant formula, and others have stepped in to fill the gap in providing such material. This provides these entities, such as online groups which are likely to have links with manufacturers of commercial formula products, with the opportunity to lead the narrative regarding formula feeding. However, the results from this research suggest that mothers need better access to such information. There are some examples of government-led campaigns, such as the national Side-by-side campaign [20] and the provincial Western Cape First Thousand Days campaign [21], which have useful websites and social media platforms that share IYCF information predominantly about breastfeeding. The accounts of these organisations have far fewer followers than corporate accounts, probably due to the sponsored posts that manufacturers of commercial products use to increase user engagement and following. Active promotion of breastfeeding is required, with marketing campaigns that counteract commercially sponsored content. Government and civil society should ensure that neutral and non-commercial information on IYCF, including about formula feeding, is made available online and at the point of sale of such products (such as in-store at supermarkets and pharmacies). More financial resources and capacity are needed for civil society and government to have a wider reach so that consumers can receive evidence-based IYCF information that is free from commercial influence. This could be achieved if social media companies consider reducing the advertising cost for non-profit and government organisations to be able to afford more ‘sponsored’ posts.

The results of this research show that product packaging is still a marketing strategy used by manufacturers, with participants describing how packaging can imply product superiority, create confusion around ages and stages of products, and influence consumer choice. In addition, cross-promotion is used to market infant formula (prohibited by the R991 regulations) through the marketing of products intended for children older than 36 months (not prohibited by the R991 regulations). In this way, messaging related to products for older children could be interpreted by consumers to apply to products intended for younger children and infants. This use of cross-promotion is a powerful marketing strategy that builds brand loyalty in consumers. Although South African legislation provides detailed guidance on labelling, a WHO review suggests that this is insufficient [11], which is supported by the results of this study. National legislation should be reviewed to standardise packaging with neutral colours and designs while also ensuring that clear unbiased information is provided on products.

Many participants in this study provided examples of exposure to commercial formula products and their manufacturers through social media and described belonging to various WhatsApp groups where information on infant feeding is sought or shared. No examples from WhatsApp groups were provided by participants which demonstrates that instant messaging platforms such as WhatsApp are difficult to monitor, since content is private. However, this does not stop representatives from companies joining such groups and sharing content on products that could influence mothers’ feeding practices. WhatsApp is the most popular mobile phone application in South Africa [22]. The use of social media marketing to violate the Code has been documented in South Africa [12] and other countries, with the COVID-19 pandemic being unfortunately used in an opportunistic manner [9]. The variety of new strategies used by companies has increased exponentially, in large part due to the innovative approaches that digital platforms provide. This compounds the already difficult task of national legislation oversight. It has been recommended that manufacturers of commercial formula products should be held accountable for their transgressions of the Code, with social media platforms having secondary accountability [9,12]. Recent examples of effective online monitoring have been displayed on some social media platforms. On Facebook, when a user shares content related to COVID-19, a pop-up message appears that alerts the user that the post contains such content and provides a link to a COVID-19 Information Centre [23]. The platforms that host online content, therefore, need to take some responsibility to familiarise themselves with international infant feeding guidance such as the Code, so that they can develop policies to guide users regarding the type of marketing content related to commercial formula products that is prohibited.

The digital advertising environment in general is complex and difficult to regulate. Exposure to digital marketing also requires access to the internet, a smartphone, and a level of digital literacy. It would therefore be helpful if digital media literacy training is incorporated when public health messaging approaches are developed. Strategies to monitor digital marketing in general are also required and the provision of guidance and technical support from international authorities is welcomed. Product labelling regulations require messages indicating that ‘breastfeeding is best’, but such guidance does not yet exist for IYCF information shared on digital platforms. It should be mandatory that any online content on IYCF or commercial formula products also needs to acknowledge the superiority of breastfeeding, as is done on product packaging.

### Public policy implications

Some of the new strategies used by companies to market commercial formula are not explicitly defined in national legislation such as the R991 regulations or in the Code and subsequent WHA guidance. Therefore, the expansion of what constitutes promotion needs to be regularly updated by national legislation and global guidance, to keep in line with novel marketing strategies developed by the advertising industry. As has been recommended by others, countries can choose to have legislation that goes beyond the recommendations of global guidance and that covers a wide range of marketing tactics [9]. In South Africa, it appears that regulation is required for commercial products targeted to children over 36 months in addition to strengthening the existing national legislation regulating products for infants and children under 36 months.

### Strengths and limitations

This is the first study that we know of in South Africa to use mobile phone marketing diaries as a method of documenting women’s exposure to the marketing of commercial formula products. Although a relatively small number of mothers were interviewed, rich data was obtained. Phone marketing diaries were only kept for one week and mothers may not have had an appointment at a health facility during that time, so the phone marketing diaries do not report on health facility related practices. Study participants were limited to women who had access to a smartphone and a minimum level of digital literacy. In some examples, women were not seeing marketing, so they went out to actively seek information.

## Conclusion

Women in South Africa are being exposed to a variety of marketing techniques from manufacturers and retailers of commercial formula products and are receiving inconsistent, and often commercially influenced, information on infant and young child feeding and nutrition. Government-led initiatives that provide evidence-based, up-to-date information on infant and young child feeding and nutrition that is free from commercial influence need to be strengthened and provided with adequate resources. Companies involved in the marketing of commercial formula products have a responsibility to comply with national legislation and government requires support to comprehensively monitor and evaluate legislation regulating the marketing of commercial formula products.

## Data Availability

Data is available on request.

## Paper context

The marketing of commercial formula products continues to take place despite global guidance and national legislation to restrict the promotion of breast-milk substitutes. Companies are using innovative strategies and new platforms such as the digital space to circumvent regulation and consumers report being confused and overwhelmed by the information available. Guidance should be regularly updated, and implementation requires urgent priority to protect consumers from inappropriate marketing practices thereby safeguarding optimal infant and young child feeding.
